# Myocardial fibroblast activation imaging in prediction of cardiac functional improvement of non-ischemic heart failure

**DOI:** 10.1016/j.ijcha.2025.101752

**Published:** 2025-07-20

**Authors:** Yao Su, Xin Liu, Ning Fu, Lina Dou, Qili Zhang, Jinxia Zhao, Qi Yang, Cunzhi Lu, Min-Fu Yang

**Affiliations:** a Department of Nuclear Medicine, Beijing Chaoyang Hospital, Capital Medical University, Beijing 100020, China; b Department of Radiology, Beijing Chaoyang Hospital, Capital Medical University, Beijing 100020, China; c Department of Nuclear Medicine, Xuzhou Central Hospital, Xuzhou 221009, China; d Department of Radiology, Xuzhou Central Hospital, Xuzhou 221009, China; e Department of Cardiology, Cardiovascular Imaging Center, Beijing Chaoyang Hospital, Capital Medical University, Beijing 100020, China; f Department of Ultrasound, Xuzhou Central Hospital, Xuzhou 221009, China; g Key Laboratory of Medical Engineering for Cardiovascular Disease, Ministry of Education, Beijing 100020, China; h Laboratory for Clinical Medicine, Capital Medical University, Beijing 100020, China

**Keywords:** ^99m^Tc-HFAPi, CMR, Non-ischemic heart failure, Myocardial fibroblast activation, Cardiac function

## Abstract

**Background:**

Activated myocardial fibroblasts are key contributors to cardiac fibrosis and drive progression toward heart failure (HF). This study aimed to investigate the characteristics of myocardial fibroblast activation imaging and its predictive value for improved cardiac function in non-ischemic HF.

**Methods:**

This double-center prospective study enrolled 38 patients who underwent ^99m^Tc-labeled-hydrazinonicotinamide-fibroblast activation protein inhibitor-04 (^99m^Tc-HFAPi) and cardiac magnetic resonance (CMR) imaging. For comparison, 18 healthy volunteers were recruited to undergo ^99m^Tc-HFAPi imaging as controls, while another 18 controls were selected from the CMR database. Myocardial ^99m^Tc-HFAPi activity was quantified by intensity, extent, and amount. CMR-derived T1 values, extracellular volume (ECV), and strain were analyzed. Baseline and follow-up echocardiographic data were used to evaluate improved cardiac function.

**Results:**

All patients exhibited intense but inhomogeneous ^99m^Tc-HFAPi uptake in the left ventricular (LV) myocardium, and the intensity was higher than that of controls (4.1 ± 1.8 vs. 1.2 ± 0.1, *p* < 0.001). LV ^99m^Tc-HFAPi amount negatively correlated with LVEF (r = -0.43, *p* = 0.008). At the segmental level, abnormal ^99m^Tc-HFAPi uptake was present in 79.8 % of segments, exceeding the prevalence of increased native T1 values (66.1 %) (p < 0.001). At median follow-up of 3 months, patients without improved cardiac function demonstrated significantly higher intensity (5.0 ± 2.0 vs. 3.5 ± 1.6, *p* = 0.022).

**Conclusion:**

Patients with non-ischemic HF showed intense but heterogeneous ^99m^Tc-HFAPi activity. The ^99m^Tc-HFAPi activity was negatively correlated with baseline cardiac systolic function and associated with poor improvement in cardiac function during follow-up.

## 1 Introduction

Heart failure (HF) remains a major global public health challenge, with non-ischemic etiologies contributing to approximately 50 % of all cases and driven by complex mechanisms such as ventricular remodeling, abnormal tissue infiltration, and chronic inflammation [[1], [2], [3]]. In non-ischemic HF, activation of myocardial fibroblasts plays a central role in disease progression, contributing to fibrotic repair but excessive ECM accumulation resulting in progressive systolic and diastolic dysfunction [4,5]. Therefore, assessing myocardial fibroblast activation is clinically critical for precisely quantifying the extent of myocardial injury and repair, guiding therapeutic interventions, and evaluating prognosis.

Cardiac magnetic resonance (CMR) is a non-invasive cornerstone for evaluating myocardial function, tissue characteristics, and fibrosis in HF patients [[6], [7], [8]]. Advanced CMR techniques such as T1 mapping [9] and extracellular volume (ECV) quantification [10] detect diffuse myocardial fibrosis and ECM expansion. However, these methods predominantly capture late-stage structural remodeling, as ECM deposition represents irreversible, established pathological changes. This limitation emphasizes the urgent need for early detection and timely interventions to prevent adverse outcomes by addressing fibrosis before it progresses to irreversible stages.

Fibroblast activation protein (FAP), a type II transmembrane glycoprotein with collagenase activity, serves as a specific marker of fibroblast activation [11]. While positron emission tomography/computed tomography (PET/CT) imaging using radiolabeled FAP inhibitor (FAPI) effectively detects early active myocardial fibrosis in HF [[12], [13], [14]], its clinical use is limited by high costs and restricted accessibility [15]. Alternatively, single-photon emission computed tomography/computed tomography (SPECT/CT) with the ^99m^Tc-labeled-hydrazinonicotinamide-fibroblast activation protein inhibitor-04 (^99m^Tc-HFAPi) has emerged as a cost-effective method for diagnosing active myocardial fibrosis in hypertension and myocarditis [16,17]. However, its application in non-ischemic HF and its potential as a prognostic marker remain underexplored.

Therefore, this prospective study aimed to assess the feasibility of ^99m^Tc-HFAPi SPECT/CT in patients with non-ischemic HF, evaluate the characteristics of myocardial fibroblast activation, and investigate its clinical potential in predicting improved cardiac function. These objectives collectively sought to establish a scientific foundation for developing more cost-effective and accessible imaging technologies tailored to HF management.

## 2 Methods

### 2.1 Study population

This prospective, double-center study was approved by the Institutional Ethics Committees of Beijing Chaoyang Hospital (2022-ke-535) and Xuzhou Central Hospital (XZXY-Lk-20230406–055), respectively. Written informed consent was obtained from all participants before enrollment. HF was diagnosed according to established criteria [2], requiring the presence of three key components: (1) Clinical manifestations: typical symptoms (e.g., dyspnea, ankle edema, and fatigue) and/or signs (e.g., elevated jugular vein pressure, pulmonary rales, and peripheral edema) caused by cardiac structural and/or functional abnormalities. (2) Biomarker elevation: elevated levels of natriuretic peptides (e.g., N-terminal pro-B type natriuretic peptide [NT-proBNP] ≥ 125 pg/mL and/or B-type natriuretic peptide [BNP] ≥ 35 pg/mL). (3) Cardiac structural or functional abnormalities: echocardiography or CMR evidence of structural abnormalities (e.g., left ventricular [LV] hypertrophy and/or left atrial [LA] enlargement) and/or diastolic dysfunction (e.g., E/e′ ≥ 13 and mean e′ velocity of septal and lateral walls < 9 cm/s). Furthermore, patients with ischemic heart disease were excluded based on coronary angiography, computed tomography, or nuclear myocardial perfusion imaging.

### 2.2 Normal control population

The health control group consisted of two components: (1) 18 healthy volunteers prospectively recruited (61.1 % [11/18] men; mean age 36.3 ± 9.3 years) who underwent ^99m^Tc-HFAPi SPECT/CT imaging; (2) an additional 18 healthy individuals (61.1 % [11/18] men; 30.4 ± 4.9 years) selected from the magnetic resonance imaging database at Beijing Chaoyang Hospital. The inclusion criteria for the healthy control group included: (1) age- and sex- matched to the patients; (2) no documented history of cardiovascular disease and no abnormalities in cardiac structure and/or function; (3) no history of malignancy. All participants underwent ^99m^Tc-HFAPi SPECT/CT and CMR imaging, with acquisition protocols described in the supplementary materials.

### 2.3 ^99m^Tc-HFAPi image interpretation

^99m^Tc-HFAPi was radiolabeled following the previously described protocol [18]. ^99m^Tc-HFAPi images were independently evaluated by three experienced nuclear physicians (YS, MFY, and CZL), blinded to the clinical information and CMR data. Disagreements were resolved by consensus. Image analysis includes both qualitative and quantitative assessments.

Qualitative analysis involved visual assessment of ^99m^Tc-HFAPi uptake in LV myocardium. Abnormal ^99m^Tc-HFAPi uptake was defined as LV myocardium activity exceeding the adjacent mediastinal blood pool background.

Quantitative analysis encompassed both global and segmental levels. At the global level, the myocardial regions of interest (ROIs) were manually delineated along the anatomical boundaries of the LV in transaxial fused SPECT/CT images. The highest maximum standard uptake value (SUVmax) across all layers was selected as the myocardial uptake index. A 10 mm diameter circular ROI was placed in the LA cavity at the level of the right lower pulmonary vein to measure the background mean standard uptake value (SUVmean). The target-to-background ratio (TBR) was calculated by dividing the LV myocardial SUVmax by the LA SUVmean, representing global uptake intensity. The raw data of ^99m^Tc-HFAPi images were processed using the QPS software (version 3.1, Cedars-Sinai Medical Center, CA, USA) to generate the polar plot. The percentage of myocardial pixels with uptake exceeding a predefined 40 % threshold [19] to the global myocardial pixel area of the LV was calculated as the ^99m^Tc-HFAPi%, reflecting the extent of ^99m^Tc-HFAPi uptake. Finally, the ^99m^Tc-HFAPi amount was calculated by the product of the ^99m^Tc-HFAPi% and TBR [17,19].

At the segmental level, the LV was divided into 17 segments according to the American Heart Association 17-segment model. The QPS software automatically calculated the percentage of average uptake counts of each segment to the maximum uptake count of LV [17,19]. The product of this percentage and global LV TBR was calculated to indicate the segmental LV ^99m^Tc-HFAPi intensity. A segment was defined as ^99m^Tc-HFAPi-positive if its TBR exceeded the mean global TBR of a healthy control group by 2 standard deviations (SD). The apical segment was excluded from the intersegment comparison analysis of ^99m^Tc-HFAPi activity and CMR.

### 2.4 Transthoracic echocardiography (TTE)

Patients underwent TTE examination at baseline and during follow-up. Improved cardiac function was defined according to the 2022AHA/ACC/HFSA guideline for the management of HF [20], requiring fulfillment of any of the following criteria: (1) baseline LVEF ≤ 40 % with a follow-up LVEF > 40 %; (2) baseline LVEF ranges from 41 % to 49 % with a follow-up LVEF > 50 % at follow-up; or (3) baseline LVEF ≥ 50 % with documented improvement in diastolic function at follow-up. All assessments were performed by experienced echocardiographers blinded to clinical data.

### 2.5 Statistical analysis

All statistical analysis was performed using SPSS Statistics 26.0 (IBM Corp., Armonk, NY, USA) and GraphPad Prism 8.0.2 (GraphPad Software, San Diego, CA, USA). Continuous variables were expressed as mean ± SD or medians with interquartile range (IQR), and categorical variables were expressed as frequencies or percentages. Patients were divided into two groups using the median ^99m^Tc-HFAPi amount as the cutoff value to display the basic characteristics. Patients with a ^99m^Tc-HFAPi amount greater than or equal to the median ^99m^Tc-HFAPi amount were defined as the high ^99m^Tc-HFAPi amount group; the other patients were categorized as the low ^99m^Tc-HFAPi amount group. Normality was checked by using the Kolmogorov-Smirnov test. Variables between the groups were compared using the *t*-test, Mann-Whitney *U* test, chi-square test, Fisher exact test, or Wilcoxon test depending on the nature of the data. Spearman's rank correlation coefficient was used to explore the relationship between LV ^99m^Tc-HFAPi intensity and CMR parameters as well as laboratory examination. *p* values of less than 0.05 were considered statistically significant.

## 3 Results

### 3.1 Patients’ characteristics

Patients’ characteristics are presented in Table 1, Table 2. 38 eligible patients were enrolled, with a mean age of 43.9 ± 15.3 years and a male predominance (26/38, 68.4 %). Of these patients, 65.8 % (25/38) were classified as HFrEF, and 34.2 % (13/38) were HFpEF. Among non-ischemic etiologies, the primary contributors were dilated cardiomyopathy (16/38, 42.1 %), acute myocarditis (9/38, 23.7 %), and hypertensive heart disease (7/38, 18.4 %). The majority of patients (36/38, 94.7 %) exhibited New York Heart Association (NYHA) functional class II or higher.

**Table 1:** Demographic characteristics of patients with non-ischemic HF.

**Characteristics**	**All patients** **(N = 38)**	**High ^99m^Tc-HFAPi amount** **(n = 19)**	**Low ^99m^Tc-HFAPi amount** **(n = 19)**	***p* value**
**Male, n (%)**	26 (68.4)	14 (73.7)	12 (63.2)	0.485
**Age, years**	43.9 ± 15.3	46.4 ± 16.6	41.5 ± 13.9	0.337
**BMI, kg/m^2^**	26.4 ± 6.0	28.1 ± 6.9	25.0 ± 4.9	0.151
**Disease course, months**	1.0 (0.9, 7.5)	3.0 (0.5, 12.0)	1.0 (1.0, 6.0)	0.525
**Cardiac risk factors**				
Hypertension	16 (42.1)	9 (47.4)	7 (36.8)	0.511
Diabetes mellitus	7 (18.4)	4 (21.1)	3 (15.8)	1.000
Dyslipidemia	8 (21.1)	2 (10.5)	6 (31.6)	0.232
Current smoker	9 (23.7)	6 (31.6)	3 (15.8)	0.447
**Medical history**				
Atrial fibrillation, n (%)	2 (4.9)	1 (4.8)	1 (5.0)	1.000
Chronic kidney disease, n (%)	9 (22.0)	5 (23.8)	4 (20.0)	1.000
New York Heart Association classification				0.384
I, n (%)	2 (5.3)	2 (10.5)	0 (0.0)	
II, n (%)	10 (26.3)	4 (21.1)	6 (31.6)	
III, n (%)	16 (42.1)	7 (36.8)	9 (47.4)	
IV, n (%)	10 (26.3)	6 (31.6)	4 (21.1)	
**Etiology**				0.494
Dilated, n (%)	16 (42.1)	9 (47.4)	7 (36.8)	
Acute myocarditis, n (%)	9 (23.7)	3 (15.8)	6 (31.6)	
Hypertensive, n (%)	7 (18.4)	4 (21.1)	3 (15.8)	
Hypertrophic, n (%)	2 (5.3)	0 (0.0)	2 (10.5)	
Amyloidosis, n (%)	1 (2.6)	1 (5.3)	0 (0.0)	
Patent foramen ovale, n (%)	1 (2.6)	1 (5.3)	0 (0.0)	
Unknown, n (%)	2 (5.3)	1 (5.3)	1 (5.3)	
**Classification according to LVEF**				**0.038**
HFrEF, n (%)	25 (65.8)	16 (84.2)	9 (47.4)	
HFpEF, n (%)	13 (34.2)	3 (15.8)	10 (52.6)	

High ^99m^Tc-labeled–hydrazinonicotinamide–fibroblast activation protein inhibitor-4 (^99m^Tc-HFAPi) amount group was defined as the ^99m^Tc-HFAPi amount of patients being greater than or equal to the median ^99m^Tc-HFAPi amount. In contrast, the other patients were defined as the low ^99m^Tc-HFAPi amount group. HF, heart failure; BMI, body mass index; LVEF, left ventricular ejection fraction; HFrEF, heart failure with reduced ejection fraction; HFpEF, heart failure with preserved ejection fraction.

**Table 2:** Imaging characteristics of patients with non-ischemic HF and controls.

**Imaging characteristics**	**High ^99m^Tc-HFAPi amount** **(n = 19)**	**Low ^99m^Tc-HFAPi amount** **(n = 19)**	**Controls** **(n = 18)**	***p* value**
**CMR parameters**				
LVEF, %	28.7 ± 15.2^§^*	43.0 ± 17.6^#^*	59.2 ± 2.7^§#^	**< 0.001**
LVEDVI, mL/m^2^	112.4 (93.6, 140.5)*	90.3 (70.7, 128.7)*	55.4 (50.3, 57.2)^§#^	**< 0.001**
LVESVI, mL/m^2^	80.9 (60.3, 127.2)*	47.4 (29.0, 88.0)*	21.1 (20.0, 23.8)^§#^	**< 0.001**
SVI, mL/m^2^	32.8 ± 12.6*	37.4 ± 12.3	45.4 ± 6.5^#^	**0.036**
CI, L/(min·m^2^)	2.5 ± 0.9*	2.6 ± 0.9	3.2 ± 0.6^#^	0.200
LV mass, g	154.0 (129.7, 201.6)*	121.5 (102.5, 159.7)*	81.9 (76.8, 86.4)^§#^	**< 0.001**
Native T1	1351.0 ± 105.0*	1319.3 ± 98.8*	1212.8 ± 22.8^§#^	**< 0.001**
ECV	34.4 ± 8.5*	34.5 ± 7.2*	26.1 ± 0.8^§#^	**< 0.001**
Longitudinal strain	−7.9 ± 3.7^§^*	−11.9 ± 5.7^#^*	−18.9 ± 2.0^§#^	**< 0.001**
Radial strain	10.5 ± 7.2^§^*	18.6 ± 10.8^#^*	34.9 ± 4.2^§#^	**0.001**
Circumferential strain	−7.8 ± 4.1^§^*	−12.2 ± 5.6^#^*	−19.9 ± 2.0^§#^	**0.001**
**^99m^Tc-HFAPi imaging**				
TBR	5.1 ± 1.7^§^*	3.1 ± 1.4^#^*	1.2 ± 0.1^§#^	**< 0.001**
Extent, %	63.4 ± 19.3	63.8 ± 25.1	**–**	0.956
Amount	3.0 ± 0.7^§^	1.7 ± 0.5^#^	**–**	**< 0.001**
No. of patients with RV uptake, %	14 (73.7)	10 (52.6)	**–**	0.179
No. of patients with atrial uptake, %	13 (68.4)	5 (26.3)	**–**	**0.009**

High ^99m^Tc-labeled–hydrazinonicotinamide–fibroblast activation protein inhibitor-4 (^99m^Tc-HFAPi) amount group was defined as the ^99m^Tc-HFAPi amount of patients being greater than or equal to the median ^99m^Tc-HFAPi amount. In contrast, the other patients were defined as the low ^99m^Tc-HFAPi amount group. HF, heart failure; CMR, cardiac magnetic resonance; LVEF, left ventricular ejection fraction; LVEDVI, left ventricular end-diastolic volume index; LVESVI, left ventricular end-systolic volume index; SVI, stroke volume index; CI, cardiac index; ECV, extracellular volume; TBR, target-to-background ratio; RV, right ventricle. ^#^*p* < 0.05 versus the high ^99m^Tc-HFAPi amount, ^§^*p* < 0.05 versus the low ^99m^Tc-HFAPi amount, *p < 0.05 versus controls.

### 3.2 Characteristics of cardiac ^99m^Tc-HFAPi activity

**Fig. 1:**
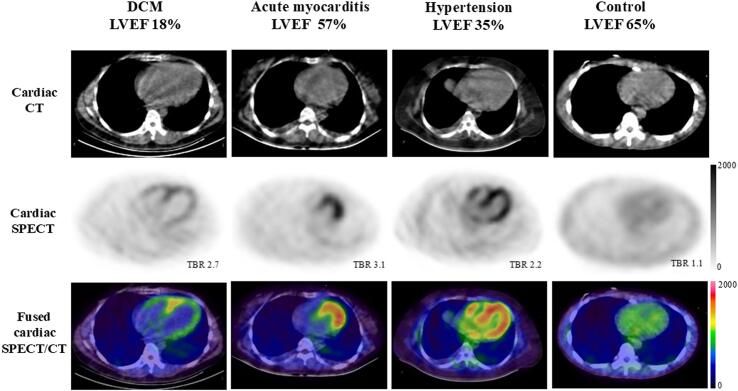
**Detailed ^99m^Tc-HFAPi images of the patients with non-ischemic HF and control.** CT (first row), SPECT (second row), and fused SPECT/CT (third row) images in patients caused by different etiologies (DCM [first column], acute myocarditis [second column], hypertension [third column]) and control (fourth column). Intense but heterogeneous ^99m^Tc-HFAPi uptake was observed in the LV myocardium of patients, whereas ^99m^Tc-HFAPi uptake was not observed in the control. ^99m^Tc-HFAPi, ^99m^Tc-labeled–hydrazinonicotinamide-fibroblast activation protein inhibitor-04; SPECT/CT, single photon emission computed tomography/computed tomography; DCM, dilated cardiomyopathy; LV, left ventricle.

All patients exhibited intense but heterogeneous ^99m^Tc-HFAPi activity in the LV, with a mean extent of ^99m^Tc-HFAPi-avid myocardium of 63.6 % ± 22.0 %. In contrast, no abnormal myocardial ^99m^Tc-HFAPi uptake was observed in control subjects (Fig. 1). Notably, the LV intensity of ^99m^Tc-HFAPi in patients was significantly elevated compared to controls (4.1 ± 1.8 vs. 1.2 ± 0.1, *p* < 0.001). Additionally, 73.7 % demonstrated ^99m^Tc-HFAPi activity in the right ventricle, while 13 patients showed ^99m^Tc-HFAPi uptake in the atrium. Importantly, none of these patients had a history of atrial fibrillation (Table 2). The imaging characteristics of three patients with Coronavirus Disease 2019-related myocarditis have been previously reported [17].

Demographic characteristics and cardiac risk factors showed no significant intergroup differences between patients with high and low ^99m^Tc-HFAPi amounts (Table 1). However, compared with the low ^99m^Tc-HFAPi amount group, the high ^99m^Tc-HFAPi amount group demonstrated a higher prevalence of HFrEF (84.2 % vs. 47.4 %, *p* = 0.038), elevated TBR (5.1 ± 1.7 vs. 3.1 ± 1.4, *p* < 0.001), and increased ^99m^Tc-HFAPi amount (3.0 ± 0.7 vs. 1.7 ± 0.5, *p* < 0.001) (Table 1, Table 2). Additionally, the high ^99m^Tc-HFAPi amount group demonstrated a significantly higher prevalence of abnormal ^99m^Tc-HFAPi uptake in the atrium (68.4 % vs. 26.3 %, *p* = 0.009) (Table 2).

### 3.3 Cardiac ^99m^Tc-HFAPi activity versus CMR

Compared with healthy controls, patients exhibited statistically significant differences in CMR structural and functional parameters (all *p* < 0.05) (Table 2). The LV ^99m^Tc-HFAPi amount was negatively correlated with LVEF (r = -0.43, *p* = 0.008) and radial strain (r = -0.53, *p* = 0.003), while positively associated with circumferential strain (r = 0.53, *p* = 0.003) and longitudinal strain (r = 0.48, *p* = 0.007).

**Fig. 2:**
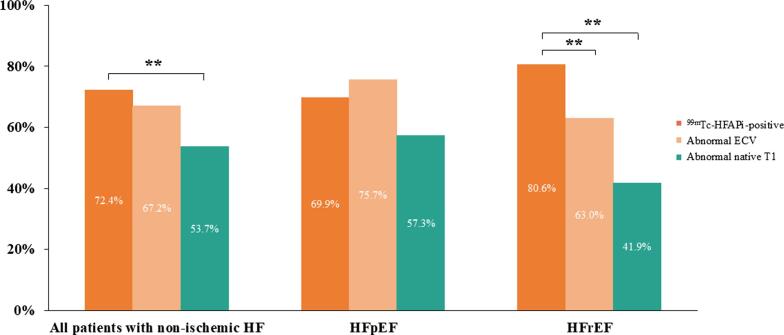
**The proportion of segments with ^99m^Tc-HFAPi-positive and abnormal CMR parameters in patients.** Statistical significance was assessed by the chi-square test. ** indicates *p* < 0.001. ^99m^Tc-HFAPi, ^99m^Tc-labeled–hydrazinonicotinamide-fibroblast activation protein inhibitor-04; CMR, cardiac magnetic resonance; ECV, extracellular volume; HFrEF, heart failure with reduced ejection fraction; HFpEF, heart failure with preserved ejection fraction.

**Fig. 3:**
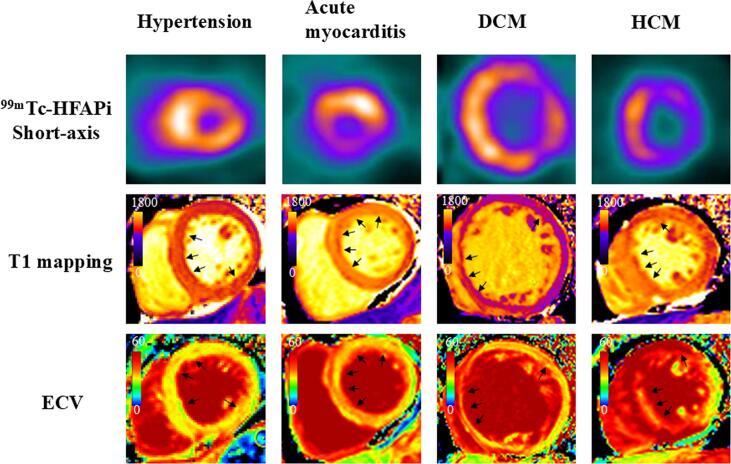
**Detailed ^99m^Tc-HFAPi and CMR images of the patients.** Short-axis images of ^99m^Tc-HFAPi (first column), T1 mapping (second column), T2 mapping (third column), and ECV(fourth column) of four patients with non-ischemic HF. Intense but heterogeneous ^99m^Tc-HFAPi uptake was observed in the LV, along with increased native T1 values (1,306.0 [1,246.5–1,430.4] ms) and increased ECV (32.0% [32.0–339.0]). An increase in T2 values (43.0 [41.5–50.0] ms) was observed in some segments. The black arrows show the abnormal area. ^99m^Tc-HFAPi, ^99m^Tc-labeled–hydrazinonicotinamide-fibroblast activation protein inhibitor-04; SA, short axis; ECV, extracellular volume; LV, left ventricle; CMR, cardiac magnetic resonance.

In addition, the segmental ^99m^Tc-HFAPi intensity demonstrated positive correlations with native T1 values (r = 0.26, *p* = 0.002) and ECV (r = 0.24, *p* = 0.005) (Table 3). At the segmental level, the proportion of ^99m^Tc-HFAPi-positive segments was significantly higher than the proportion of segments with elevated native T1 values (79.8 % vs. 66.1 %, *p* < 0.001), as demonstrated in Fig. 2. Representative images of ^99m^Tc-HFAPi and CMR findings are illustrated in Fig. 3.

**Table 3:** Correlation of LV ^99m^Tc-HFAPi intensity and CMR parameters in patients with non-ischemic HF.

	**LV ^99m^Tc-HFAPi intensity**	
	**r**	***p* value**
**Global level**		
LVEF, %	**−0.341**	**0.036**
LVEDVI, mL/m^2^	0.222	0.239
LVESVI, mL/m^2^	0.224	0.233
LV mass, g	0.271	0.147
SVI, mL/m^2^	−0.320	0.233
CI, L/(min·m^2^)	−0.217	0.250
Native T1	0.237	0.329
ECV	0.332	0.165
Longitudinal strain	**0.588**	**0.001**
Radial strain	**−0.456**	**0.011**
Circumferential strain	**0.481**	**0.007**
**Segmental level**		
Native T1	**0.264**	**0.002**
ECV	**0.239**	**0.005**
Longitudinal strain	**0.330**	**< 0.001**
Radial strain	**−0.221**	**0.008**
Circumferential strain	**0.197**	**0.018**

LV, left ventricle; ^99m^Tc-HFAPi, ^99m^Tc-labeled–hydrazinonicotinamide–fibroblast activation protein inhibitor-4; CMR, cardiac magnetic resonance; HF, heart failure; LVEF, left ventricular ejection fraction; LVEDVI, left ventricular end-diastolic volume index; LVESVI, left ventricular end-systolic volume index; SVI, stroke volume index; CI, cardiac index; ECV, extracellular volume.

### 3.4 Comparison among major non-ischemic HF etiologies

Recognizing the potential influence of underlying cause, we compared patients with three prevalent non-ischemic HF etiologies: dilated cardiomyopathy (HF-DCM, n = 16), acute myocarditis (HF-myocarditis, n = 9), and hypertensive heart disease (HF-HT, n = 7) (Table 4). No significant differences were observed among the three groups in baseline demographic characteristics, most traditional cardiovascular risk factors, and history of atrial fibrillation. However, the prevalence of hypertension (100 % vs. 31.3 % vs. 22.2 %, *p* = 0.003) and chronic kidney disease (71.4 % vs. 6.3 % vs. 11.1 %, *p* = 0.004) was significantly higher in the HF-HT group compared to the HF-DCM and HF-myocarditis groups. In addition, all HF-DCM patients had HFrEF, the HF-myocarditis group was predominantly HFpEF (88.9 %), while the HF-HT group showed a mixed phenotype (HFrEF 57.1 %, HFpEF 42.9 %) (*p* < 0.001).

**Table 4:** Comparison among the major etiologies of non-ischemic HF.

**Characteristics**	**HF-DCM** **(n = 16)**	**HF-myocarditis** **(n = 9)**	**HF-HT** **(n = 7)**	***p* value**
**Male, n (%)**	11 (68.8)	5 (55.6)	5 (71.4)	0.791
**Age, years**	47.8 ± 12.6	40.2 ± 19.9	40.3 ± 12.9	0.376
**BMI, kg/m^2^**	27.1 ± 7.6	23.1 ± 3.8*	30.0 ± 4.4^§^	0.100
**Disease course, months**	1.5 (1.0, 12.0)	0.5 (0.3, 1.0)	1.0 (1.0, 6.0)	0.325
**Cardiac risk factors**				
Hypertension	5 (31.3)	2 (22.2)	7 (100.0)	**0.003**
Diabetes mellitus	4 (25.0)	0 (0.0)	2 (28.6)	0.255
Dyslipidemia	1 (6.3)	3 (33.3)	3 (42.9)	0.067
Current smoker	5 (31.3)	2 (22.2)	2 (28.6)	1.000
**Medical history**				
Atrial fibrillation, n (%)	1 (6.3)	0 (0.0)	0 (0.0)	1.000
Chronic kidney disease, n (%)	1 (6.3)	1 (11.1)	5 (71.4)	**0.004**
**New York Heart Association** **classification**				0.186
I, n (%)	1 (6.3)	0 (0.0)	1 (14.3)	
II, n (%)	1 (6.3)	6 (66.7)	0 (0.0)	
III, n (%)	8 (50.0)	1 (11.1)	4 (57.1)	
IV, n (%)	6 (37.5)	2 (22.2)	2 (28.6)	
**Classification according to LVEF**				**< 0.001**
HFrEF, n (%)	16 (100.0)	1 (11.1)	4 (57.1)	
HFpEF, n (%)	0 (0.0)	8 (88.9)	3 (42.9)	
**CMR parameters**				
LVEF, %	23.2 ± 9.2^§^*	55.5 ± 11.9^#^*	40.6 ± 15.2^§#^	**< 0.001**
LVEDVI, mL/m^2^	129.7 (102.6, 163.0)^§^	80.5 (65.8, 90.0)^#^	118.2 (80.3, 133.0)	**0.008**
LVESVI, mL/m^2^	88.8 (75.2, 134.3)^§^	30.3 (25.2, 43.0)^#^	75.7 (36.3, 115.0)	**0.005**
SVI, mL/m^2^	31.6 ± 13.6	41.6 ± 10.9	40.3 ± 5.0	0.123
CI, L/(min·m^2^)	2.3 ± 1.0*	2.8 ± 0.9	2.9 ± 0.6^#^	0.293
LV mass, g	154.1 ± 38.8^§^	113.1 ± 21.8^#^*	182.1 ± 67.0^§^	**0.019**
Native T1	1340.4 ± 95.1	1361.7 ± 114.0*	1247.8 ± 19.2^§^	0.122
ECV	35.1 ± 6.8*	36.6 ± 5.3*	27.3 ± 3.8^§#^	**0.024**
Longitudinal strain	−7.5 ± 2.3^§^*	−15.4 ± 4.4^#^	−11.4 ± 5.2^#^	**< 0.001**
Radial strain	8.9 (5.9, 11.2)^§^*	29.3 (15.4, 32.5)^#^*	12.7 (9.6, 27.0)^§#^	**< 0.001**
Circumferential strain	−7.3 (−8.6, −5.0)^§^*	−18.0 (−19.3, −11.3)^#^*	−9.6 (−17.0, −7.5)^§#^	**< 0.001**
**^99m^Tc-HFAPi imaging**				
TBR	4.8 ± 2.2^§^*	2.9 ± 0.6^#^	3.2 ± 1.5^#^	**0.025**
Extent, %	61.7 ± 21.3	68.2 ± 17.4	78.4 ± 18.2	0.189
Amount	2.6 ± 0.9	2.0 ± 0.6	2.3 ± 0.6	0.166
No. of patients with RV uptake, %	10 (62.5)	6 (66.7)	5 (71.4)	1.000
No. of patients with atrial uptake, %	11 (68.8)	3 (33.3)	2 (28.6)	0.144

HF, heart failure; DCM, dilated cardiomyopathy; HT, hypertensive; BMI, body mass index; LVEF, left ventricular ejection fraction; HFrEF, heart failure with reduced ejection fraction; HFpEF, heart failure with preserved ejection fraction; CMR, cardiac magnetic resonance; LVEF, left ventricular ejection fraction; LVEDVI, left ventricular end-diastolic volume index; LVESVI, left ventricular end-systolic volume index; SVI, stroke volume index; CI, cardiac index; ECV, extracellular volume; ^99m^Tc-HFAPi, ^99m^Tc-labeled–hydrazinonicotinamide–fibroblast activation protein inhibitor-4; TBR, target-to-background ratio; RV, right ventricle. ^#^*p* < 0.05 versus the HF due to dilated cardiomyopathy, ^§^*p* < 0.05 versus the HF due to myocarditis, *p < 0.05 versus the HF due to hypertensive heart disease.

CMR confirmed that the HF-DCM group demonstrated the lowest LVEF, the largest LVEDVI, and the worst strain in all directions. In contrast, the HF-myocarditis group showed the highest LVEF, the smallest ventricular volume, and relatively preserved strain. The HF-HT group was characterized by the highest LV mass (all *p* < 0.05). ECV was significantly higher in the HF-DCM and HF-myocarditis groups than in the HF-HT group (35.1 ± 6.8, 36.6 ± 5.3 vs. 27.3 ± 3.8, *p* = 0.024). ^99m^Tc-HFAPi imaging showed that myocardial fibroblast intensity (TBR) was significantly higher in the HF-DCM group than in the other two groups (4.8 ± 2.2 vs. 2.9 ± 0.6 vs. 3.2 ± 1.5, *p* = 0.025).

### 3.5 HFpEF versus HFrEF

The demographic and clinical characteristics of HFpEF (n = 13) and HFrEF (n = 25) patients are summarized in Table S1. The two groups were comparable in age, sex distribution, and cardiovascular risk factors (all *p* ˃ 0.05).

Myocardial ^99m^Tc-HFAPi SPECT/CT imaging revealed elevated ^99m^Tc-HFAPi uptake parameters in HFrEF: higher TBR (4.6 ± 1.9 vs. 3.1 ± 1.1, *p* = 0.006) and myocardial uptake amount (2.6 ± 0.8 vs. 1.9 ± 0.8, *p* = 0.011). Cardiac imaging revealed marked differences between groups. HFrEF patients had significantly elevated left ventricular end-diastolic volume index (127.5 [102.4–139.3] vs. 79.2 [61.4–82.5], *p* < 0.001) and end-systolic volume index (87.2 [72.0–124.8] vs. 30.3 [23.1–36.1], *p* < 0.001). Myocardial deformation analysis showed profound differences in strain parameters. HFrEF patients demonstrated significantly impaired longitudinal strain (−7.4 ± 2.4 vs. −15.3 ± 5.4, *p* = 0.001), radial strain (9.3 ± 3.8 vs. 25.9 ± 9.5, *p* < 0.001), and circumferential strain (−7.2 ± 2.6 vs. −16.0 ± 4.6, *p* < 0.001) compared to HFpEF patients.

### 3.6 Follow-up

**Fig. 4:**
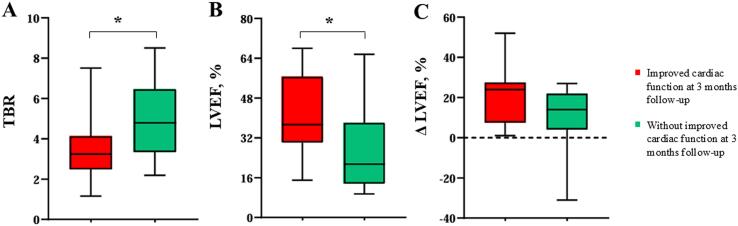
**The comparison of TBR, CMR parameters, and ΔLVEF between patients with and without improved cardiac function at 3 months of follow-up.** Statistical significance was assessed by the *t*-test. * indicates *p* < 0.05. TBR, target-to-background ration; LV, left ventricle; CMR, cardiac magnetic resonance; LVEF, left ventricular ejection fraction.

During follow-up, most commonly prescribed medications in this study were angiotensin receptor-neprilysin inhibitors (87.5 %) and β-blockers (87.5 %). Moreover, all patients diagnosed with acute myocarditis (100.0 %) were prescribed non-steroidal anti-inflammatory drugs. A total of 33 patients completed a 3-month echocardiographic follow-up assessment with a median ΔLVEF of 20.0 (6.0–25.0). Among these, 17 patients demonstrated improved cardiac function. Subgroup analysis revealed that patients without improved cardiac function exhibited significantly higher baseline TBR (5.0 ± 2.0 vs. 3.5 ± 1.6, *p* = 0.022) and lower baseline LVEF (27.8 ± 17.8 vs. 42.6 ± 15.7, p = 0.016) compared to the improved group (Fig. 4). Notably, the median ΔLVEF in the group without improved cardiac function was numerically smaller than in patients with improved cardiac function (14.0 [4.0–22.0] vs. 24.0 [7.5–27.5]), and the intergroup difference approached borderline statistical significance (*p* = 0.058). However, no significant differences in the change of LVEDVI and LVESVI were observed between the two groups (all *p* > 0.05).

## 4 Discussion

HF is a complex cardiovascular syndrome, with non-ischemic etiologies accounting for up to 50 % of all cases [20]. Myocardial fibrosis, a maladaptive response to chronic pressure overload or injury, is central to driving adverse cardiac remodeling and progression toward end-stage HF [[21], [22], [23]]. Notably, activation of myocardial fibroblasts plays a pivotal regulatory role in this process of cardiac fibrosis by transforming quiescent fibroblasts into myofibroblasts, accompanied by excessive expression of α-smooth muscle actin (α-SMA) and heightened secretion of ECM components, particularly type I and III collagen fibers. This activation both initiates and intensifies the fibrotic cascade [24]. Therefore, investigating the progression of activated myocardial fibroblasts yields dual clinical benefits, including identifying potential biomarkers for HF risk stratification and providing a theoretical framework for developing precision anti-fibrotic therapies to improve treatment efficacy and long-term outcomes.

FAP, a specific biomarker of myocardial fibroblast activation, is markly upregulated during cellular activation. Radiolabeled FAP inhibitors (e.g., ^68^Ga-FAPI, ^18^F-FAPI) combined with PET/CT enables precisely visualize and longitudinally monitor fibroblast activation [25]. Recent studies have demonstrated that HF animal models exhibited ^68^Ga-FAPI uptake in the LV myocardium that peaks early in HF progression, correlating strongly with histopathological fibroblast activation, before declining as fibrosis stabilizes into irreversible fibrotic scar tissue [[12], [13], [14], [15]]. These findings validate the clinical utility of FAPI PET/CT for early diagnosis of myocardial fibrosis and provide critical imaging evidence to define therapeutic intervention windows and assess treatment efficacy in clinical practice.

However, FAPI PET/CT has not yet undergone systematic clinical validation in non-ischemic HF and is hindered by its limited equipment availability and high examination costs. Therefore, our study is the first double-center prospective clinical trial to systematically investigate the feasibility and clinical utility of FAPI SPECT/CT in non-ischemic HF, aiming to guide cost-effective imaging development. Key findings include: (1) All patients with non-ischemic HF exhibited intense but heterogeneous ^99m^Tc-HFAPi activity in LV, with HFrEF patients having significantly higher ^99m^Tc-HFAPi activity than HFpEF. (2) ^99m^Tc-HFAPi SPECT/CT identified more affected myocardial segments than ECV and T1 mapping detected by CMR. (3) At 3 months of follow-up, patients without improved cardiac function exhibited significantly higher baseline LV ^99m^Tc-HFAPi intensity and numerically lower median ΔLVEF (borderline statistical significance).

### 4.1 LV ^99m^Tc-HFAPi activity may serve as a surrogate indicator of myocardial fibroblast activation in non-ischemic HF and a predictor of improved cardiac function

This study found significantly elevated ^99m^Tc-HFAPi uptake in the LV myocardium of non-ischemic HF patients compared to healthy controls with no abnormal uptake, indicating myocardial fibroblast activation in HF patients versus quiescence in controls [5,26]. This validates the feasibility of ^99m^Tc-HFAPi SPECT/CT in identifying early active myocardial fibrosis. Previous studies link myocardial fibroblast activation to transforming growth factor-β (TGF-β) signaling pathway, as mechanical stress-induced activation via the TGF-β1 pathway has been shown to drive fibrosis, suggesting therapeutic potential [27]. Furthermore, a significant negative correlation between LV ^99m^Tc-HFAPi activity and baseline LVEF was observed, providing novel imaging insights into disease mechanisms. Longitudinal follow-up analysis further indicated that patients without improved cardiac function exhibited significantly higher myocardial fibroblast activation, highlighting a potential dynamic association between myocardial fibroblast activation and cardiac functional recovery.

Based on these findings, we propose that early compensatory activation of myocardial fibroblasts in non-ischemic HF aims to repair and maintain cardiac structure/function via ECM regulation. However, persistent activation of myocardial fibroblasts significantly upregulates type I collagen gene expression, leading to pathological collagen deposition in the ECM. This dysregulated collagen accumulation progressively stiffens the myocardium, destabilizing ventricular geometry and culminating in compromised systolic and diastolic performance. This cascade perpetuates a self-reinforcing cycle of “fibrosis-remodeling-functional decline”, exacerbating disease progression [28].

This study was the first to validate ^99m^Tc-HFAPi SPECT/CT as an imaging tool for quantifying myocardial fibroblast activation and evaluating the improvement of cardiac systolic function in patients with non-ischemic HF. Lower levels of ^99m^Tc-HFAPi activity were associated with greater improvement in LVEF, highlighting its potential utility in assessing myocardial repair processes. Further multicenter prospective studies are needed to confirm its effectiveness in monitoring longitudinal changes in cardiac function and fibroblast activity.

### 4.2 Differential myocardial fibroblast activation between HFpEF and HFrEF

In healthy myocardium, ECM is predominantly composed of thicker type I collagen fibers, which confer tensile strength to the heart, and thinner type III collagen fibers are responsible for maintaining the elasticity of the matrix network [29]. In HFrEF, myocardial fibrosis is primarily the result of cardiomyocyte injury, which disrupts cardiomyocyte conduction pathways and damages the endocardium, thereby impairing systolic function [30]. Conversely, HFpEF is characterized by excessive collagen deposition and a relative deficiency of type III collagen, leading to increased myocardial stiffness [31].

Cardiac fibrosis arises from a dysregulated balance between ECM production and degradation, ultimately compromising systolic and diastolic cardiac function [32]. Activated myocardial fibroblasts are important participants of cardiac fibrosis in both HFpEF and HFrEF [33], however, the signaling pathways controlling cardiac fibrosis differ between these conditions. Recent studies highlight the role of TGF-β1, which is activated by multiple mediators, such as proteases, thrombospondin-1, and reactive oxygen species during cardiac injury [34]. TGF-β1 signaling promotes cardiac fibrosis by activating genes responsible for myofibroblast differentiation and ECM synthesis [35]. Interestingly, the serum concentrations of TGF-β1 protein were significantly higher in HFpEF compared to HFrEF [35], suggesting distinct pathophysiological mechanisms.

Furthermore, the spatial distribution of myocardial fibrosis exhibits distinct patterns between HFpEF and HFrEF. Perivascular fibrosis is more prominent in HFpEF, contributing to diastolic dysfunction and increased myocardial stiffness [36]. In contrast, interstitial fibrosis, which is characterized by ECM expansion surrounding cardiomyocytes, is more prevalent in HF subtypes without cardiomyocyte loss, including non-ischemic HFrEF and HFpEF [43]. Given the heart’s limited regenerative capacity, fibrotic repair is essential to maintain the myocardial structural and functional integrity following the removal of necrotic cardiomyocytes. Notably, cardiomyocyte loss is a predominant feature of HFrEF and has not been reported in HFpEF [37].

Therefore, HFrEF and HFpEF exhibit distinct differences in the composition, underlying mechanisms, and spatial distribution of cardiac fibrosis, which may account for the higher myocardial fibroblast activation levels observed in HFrEF compared to HFpEF in our study.

### 4.3 Myocardial fibroblast activation and CMR parameters

The findings of this study demonstrate a low-to-moderate positive correlation between segmental ^99m^Tc-HFAPi intensity and CMR-derived tissue characterization parameters (e.g., native T1 values and ECV). This concordance is clinically noteworthy, as CMR is recognized for its high sensitivity and specificity in identifying the myocardial pathological features such as inflammation, edema, and fibrosis [38]. The consistency of LV ^99m^Tc-HFAPi activity and CMR tissue characterization parameters indicates that ^99m^Tc-HFAPi SPECT/CT imaging has potential as a non-invasive tool to visualize myocardial injury in HFpEF. By enabling early detection of subclinical myocardial damage, ^99m^Tc-HFAPi SPECT/CT imaging could facilitate timely therapeutic intervention, thereby mitigating progressive declines in LVEF and improving clinical outcomes in this patient population.

Further analysis revealed a weak correlation between segmental ^99m^Tc-HFAPi intensity and CMR-derived parameters such as native T1 values and ECV. This observation may stem from differences in the pathophysiological stages of cardiac fibrosis that each modality preferentially detects. Specifically, CMR imaging primarily assesses the extent of myocardial fibrosis by quantifying irreversible ECM deposition within the interstitial space. In contrast, ^99m^Tc-HFAPi SPECT/CT imaging can specifically identify activated myocardial fibroblasts, enabling detection of dynamic pathological changes during the early stages of cardiac fibrosis before significant ECM synthesis occurs. Notably, segmental analysis demonstrated a significantly higher number of ^99m^Tc-HFAPi-positive segments compared to CMR-identified abnormal regions (e.g., elevated native T1 and ECV values). These findings suggest that ^99m^Tc-HFAPi SPECT/CT imaging not only enables earlier detection of the initial stages of cardiac fibrosis but also identifies a broader range of myocardial injury compared to conventional CMR tissue characterization techniques, demonstrating superior sensitivity in lesion detection.

In addition, CMR feature tracking (CMR-FT) is a computational technique that quantifies myocardial strain and strain rate using standard cine images acquired during routine clinical CMR protocols [39]. In our study, comparative analysis between ^99m^Tc-HFAPi activity and CMR-FT-derived strain metrics revealed significant correlations at both global and segmental levels, particularly for longitudinal, radial, and circumferential strain. This may be because in pathological conditions, fibrous tissue gradually replaces normal myocardial tissue. Since the fibrous tissue itself is much less elastic and contractile than normal myocardium, this structural change leads to increase ^99m^Tc-HFAPi uptake. At the same time, the presence of fibrous tissue greatly restricts the normal deformation of the myocardium, resulting in a significant reduction in myocardial strain capacity. These findings suggest that ^99m^Tc-HFAPi uptake may reflect underlying myocardial dysfunction, potentially capturing early-stage fibrotic remodeling that contributes to reduced strain in non-ischemic HF patients. Notably, the observed associations highlight a mechanistic link between myocardial fibroblast activation (detected by ^99m^Tc-HFAPi SPECT/CT) and impaired myocardial deformation (assessed by CMR-FT), underscoring the tracer’s potential as a non-invasive biomarker for subclinical structural and functional changes in HF.

### 4.4 Differences in myocardial ^99m^Tc-HFAPi uptake among DCM, acute myocarditis, and HF-HT patients

Wang et al. reported that in patients with different subtypes of non-ischemic cardiomyopathy (NICM), the uptake of ^68^Ga-FAPI in the LV showed uneven elevation, and significantly different ^68^Ga-FAPI uptake could be observed within the same type of disease [40]. In contrast, our study employed novel single-photon imaging agents and conducted a subgroup analysis, specifically comparing the ^99m^Tc-HFAPi uptake among the three main causes of non-ischemic HF. Our results indicate that compared with patients with acute myocarditis and HF-HT, patients with DCM demonstrated significantly higher myocardial ^99m^Tc-HFAPi uptake, suggesting that different etiologies lead to different patterns of fibroblast activation. This hierarchical structure of ^99m^Tc-HFAPi uptake may reflect the pathophysiological differences associated with different etiologies. In DCM, chronic mechanical and neurohormonal stress drive continuous fibroblast activation, which is consistent with the characteristic of diffuse fibrosis in DCM. Moreover, DCM is a heterogeneous disease that can manifest as various non-ischemic disease forms, such as gene mutations, metabolic and endocrine disorders, as well as exposure to alcohol, drugs, and toxins [41]. The overall ^99m^Tc-HFAPi uptake in patients with acute myocarditis is relatively low. This could be attributed to the fact that, during the acute phase (less than 3 months), the early inflammatory process overshadows the fibroblast-mediated fibrosis. Similarly, the reduced ^99m^Tc-HFAPi uptake in patients with HF-HT may be due to its interstitial fibrosis pattern [42], rather than the extensive myocardial remodeling typically seen in DCM.

## 5 Limitations

The study has some limitations. First, the small sample size limits statistical power, necessitating future multicenter trials with larger cohorts. Second, although the study enrolled a cohort of non-ischemic HF patients encompassing different LVEF subtypes (HFpEF and HFrEF), the subgroup distribution was imbalanced, which may have weakened the statistical power of subgroup analyses. Third, among the enrolled patients, neither diagnostic biopsies nor surgical resections were routinely carried out either before or after the time of ^99m^Tc-HFAPi imaging. As a result, we were unable to obtain heart tissue samples corresponding to the imaging time points for FAP immunohistochemical analysis. Fourth, non-ischemic HF has complex etiologies (e.g., acute myocarditis, DCM, hypertensive heart disease), and the fibrotic pathological features may differ significantly among different etiologies. Fifth, considering that the 3-month follow-up in this pilot study is a bit short, the short observational window and the low incidence of major adverse cardiovascular events (MACE) in non-ischemic HF patients precluded definitive evaluation of whether ^99m^Tc-HFAPi SPECT/CT imaging adds incremental prognostic value. To develop clinically useful prognostic models, future work should include longer follow-up for long-term MACE, larger multi-center cohorts, and machine learning algorithms to stratify risk by etiology and fibrosis severity.

## 6 Conclusion

This study demonstrates that myocardial fibroblasts in patients with non-ischemic HF exhibit heterogeneous degrees of activation, and the activity of myocardial fibroblast activation is associated with poorer improvement in cardiac function at 3-month follow-up. In addition, ^99m^Tc-HFAPi SPECT/CT imaging detects a broader extent of myocardial injury than CMR tissue characterization techniques, suggesting its potential for identifying a wider range of affected myocardial regions.

## CRediT authorship contribution statement

**Yao Su:** Writing – original draft, Formal analysis, Data curation. **Xin Liu:** Writing – original draft, Formal analysis, Data curation. **Ning Fu:** Formal analysis, Data curation. **Lina Dou:** Formal analysis, Data curation. **Qili Zhang:** Formal analysis, Data curation. **Jinxia Zhao:** Formal analysis, Data curation. **Qi Yang:** Writing – review & editing, Formal analysis, Data curation. **Cunzhi Lu:** Writing – review & editing, Formal analysis, Data curation. **Min-Fu Yang:** Writing – review & editing, Formal analysis, Conceptualization.

## Funding

This work was supported by the 10.13039/501100012166National Key Research and Development Program of China (grant numbers 2021YFF0501400 and 2021YFF0501401), Beijing Hospitals Authority Clinical Medicine Development of Special Funding Support (ZYLX202105), and 10.13039/501100001809National Natural Science Foundation of China (82025018).

## Declaration of competing interest

The authors declare that they have no known competing financial interests or personal relationships that could have appeared to influence the work reported in this paper.
