# Supplementary material for: Myocardial fibroblast activation imaging in prediction of cardiac functional improvement of non-ischemic heart failure
Source: Int J Cardiol Heart Vasc. 2025 Jul 20;60:101752. doi: 10.1016/j.ijcha.2025.101752 (PMC12490530; doi:10.1016/j.ijcha.2025.101752)
Supplement: Supplementary Data 1 [file mmc1.docx]

**Supplemental Table 1.** Comparative analysis of clinical characteristics between patients with HFpEF and HFrEF

| **Characteristics** | **HFpEF**  **(n=13)** | **HFrEF**  **(n=25)** | ***p* value** |
| --- | --- | --- | --- |
| **Male, n (%)** | 8 (61.5) | 18 (72.0) | 0.510 |
| **Age, years** | 38.0 ± 14.8 | 47.0 ± 14.9 | 0.084 |
| **BMI, kg/m^2^** | 25.1 ± 5.6 | 27.1 ± 6.2 | 0.371 |
| **Cardiac risk factors** |  |  |  |
| Hypertension | 4 (30.8) | 12 (48.0) | 0.490 |
| Diabetes mellitus | 0 (0.0) | 7 (28.0) | 0.072 |
| Dyslipidemia | 3 (23.1) | 5 (20.0) | 1.000 |
| Current smoker | 2 (15.4) | 7 (28.0) | 0.456 |
| **Medical history** |  |  |  |
| Atrial fibrillation, n (%) | 0 (0.0) | 2 (8.0) | 0.538 |
| Chronic kidney disease, n (%) | 3 (23.1) | 5 (20.0) | 1.000 |
| New York Heart Association  classification |  |  | 0.067 |
| I, n (%) | 1 (7.7) | 1 (4.0) |  |
| II, n (%) | 7 (53.8) | 3 (12.0) |  |
| III, n (%) | 2 (15.4) | 14 (56.0) |  |
| IV, n (%) | 3 (23.1) | 7 (28.0) |  |
| **Etiology** |  |  | 0.185 |
| Hypertensive, n (%) | 3 (23.1) | 4 (16.0) |  |
| Dilated, n (%) | 0 (0.0) | 16 (64.0) |  |
| Hypertrophic, n (%) | 1 (7.7) | 1 (4.0) |  |
| Acute myocarditis, n (%) | 8 (61.5) | 1 (4.0) |  |
| Amyloidosis, n (%) | 0 (0.0) | 1 (4.0) |  |
| Patent foramen ovale, n (%) | 0 (0.0) | 1 (4.0) |  |
| Unknown, n (%) | 1 (7.7) | 1 (4.0) |  |
| **TBR** | 3.1 ± 1.1 | 4.6 ± 1.9 | **0.006** |
| **Extent, %** | 64.3 ± 24.6 | 63.2 ± 21.2 | 0.878 |
| **Amount** | 1.9 ± 0.8 | 2.6 ± 0.8 | **0.011** |
| **LVEF, %** | 57.5 ± 5.2 | 24.6 ± 9.5 | **< 0.001** |
| **LVEDVI, mL/m^2^** | 79.2 (61.4, 82.5) | 127.5 (102.4, 139.3) | **< 0.001** |
| **LVESVI, mL/m^2^** | 30.3 (23.1, 36.1) | 87.2 (72.0, 124.8) | **< 0.001** |
| **SVI** | 41.8 ± 10.3 | 32.0 ± 12.3 | **0.040** |
| **CI** | 2.9 ± 0.8 | 2.4 ± 0.9 | 0.167 |
| **LV mass, g** | 106.9 (101.3, 141.8) | 155.1 (123.3, 193.3) | **0.019** |
| **Native T1** | 1338.4 ± 121.6 | 1318.0 ± 77.7 | 0.674 |
| **ECV** | 34.7 ± 6.9 | 34.2 ± 8.1 | 0.889 |
| **Longitudinal strain** | -15.3 ± 5.4 | -7.4 ± 2.4 | **0.001** |
| **Radial strain** | 25.9 ± 9.5 | 9.3 ± 3.8 | **< 0.001** |
| **Circumferential strain** | -16.0 ± 4.6 | -7.2 ± 2.6 | **< 0.001** |

HFpEF, heart failure with preserved ejection fraction; HFrEF, heart failure with reduced ejection fraction; BMI, body mass index; TBR, target-to-background ratio; LVEF, left ventricular ejection fraction; LVEDVI, left ventricular end-diastolic volume index; LVESVI, left ventricular end-systolic volume index; SVI, stroke volume index; CI, cardiac index; ECV, extracellular volume.
